# Correspondence

**Published:** 1864-05

**Authors:** Julius Homberger

**Affiliations:** Editor American Journal of Ophthalmology; 39 West 23d Street, New York


### CORRESPONDENCE

39 *West* 23*d Street, New York,*
*April* 21st, 1864.

To the Editor of the Buffalo Medical and Surgical Journal:

My Dear Doctor:—I have just received your Journal, and was glad to see that the dangerous topic of Specialties and Specialists, is not dangerous enough for your independent spirit. You would not find, in many of our medical papers, the faintest allusion to this all-important question, merely because the Editor has neither the courage to attack specialists, nor to use his influence against the antiquated notions of old fogyism. You say in your editorial on Specialists that a physician may profitably cultivate one department of professional knowledge and thereby excel in that, and thus admit that specialties are conducive to the well being of the community.

You find fault with the manner of specialists to put their superiority forward, and therein you are perfectly justified, if specialists claim to belong to the regular profession. But I hold that up to the present moment those who have a right to call themselves specialists, by preliminary general education as medical men as well as by special attainments acquired afterwards, have not infringed the code of ethics of the American profession. The offensive quota of specialists are in the first place the immense number of charlatans who take up medicine like a trade, and make it their business to promise everything for the sake of money paid in advance, like the celebrated Dr. ——— in this city, late corn-cutter to Her Majesty the Queen of England, and those whom you very well characterize as "treating every known disease, when presented, and of paying special attention to them all." It is a matter of course that the latter cannot be superior to an honest general practitioner who does not resort to the same dodge. This latter class is represented in numerous specimens in the medical profession of large cities, and of our metropolis in particular. Such men have frequently puffed their names in every possible manner; they sometimes are the proprietors of dispensaries, and even schools, and heaven only knows of the injuries they do to patients and pupils. Their yearly catalogues of diseases treated, annual reports, contain statistical tables ($\frac{19}{20}$ cured, $\frac{1}{40}$ under treatment, and $\frac{1}{40}$ incurable,) so magnificent that they are impossible. But they manage to keep themselves in the profession nevertheless, and would vociferously condemn anybody who would merely advertise his residence in a newspaper.

As to the resolutions of the State Medical Society, they must have been formed with reference to this obnoxious class of mock-specialists, and unfortunately may be destined to prejudice the profession against necessary reforms—which I advocated some, alas! with but feeble support—at the meeting of the American Medical Association in Chicago, 1863, and in the first volume of the *American Journal of Ophthalmology*. Should those resolutions now be adopted by the Association, then you will witness in June, 1864, the breach between the profession at large and the small number of *real* specialists, which have always deserved to be an honor to medical science, and who have never violated the code of ethics.

I hope that your independence will allot a space to this lengthy epistle in the *Buffalo Medical and Surgical Journal*,

And remain very truly yours,

JULIUS HOMBERGER,
Editor American Journal of Ophthalmology.

